# Factorial Design and Optimization of Landfill Leachate Treatment Using Tannin-Based Natural Coagulant

**DOI:** 10.3390/polym11081349

**Published:** 2019-08-14

**Authors:** Tawfiq J. H. Banch, Marlia M. Hanafiah, Abbas F. M. Alkarkhi, Salem S. Abu Amr

**Affiliations:** 1 Center for Earth Sciences and Environment, Faculty of Science and Technology, Universiti Kebangsaan Malaysia, Bangi 43600, Selangor, Malaysia; 2 Centre for Tropical Climate Change System, Institute of Climate Change, Universiti Kebangsaan Malaysia, Bangi 43600, Selangor, Malaysia; 3 Universiti Kuala Lumpur, Malaysian Institute of Chemical & Bioengineering Technology (UniKL, MICET), Melaka 78000, Malaysia

**Keywords:** landfill, leachate treatment, tannin, coagulation, removal efficiency, heavy metals

## Abstract

In this study, tannin-based natural coagulant was used to treat stabilized landfill leachate. Tannin modified with amino group was utilized for the treatment process. Central composite design (CCD) was used to investigate and optimize the effect of tannin dosage and pH on four responses. The treatment efficiency was evaluated based on the removal of four selected (responses) parameters; namely, chemical oxygen demand (COD), color, NH_3_–N and total suspended solids (TSS). The optimum removal efficiency for COD, TSS, NH_3_–N and color was obtained using a tannin dosage of 0.73 g at a pH of 6. Moreover, the removal efficiency for selected heavy metals from leachate; namely, iron (Fe^2+^), zinc (Zn^2+^), copper (Cu^2+^), chromium (Cr^2+^), cadmium (Cd^2+^), lead (Pb^2+^), arsenic (As^3+^), and cobalt (Co^2+^) was also investigated. The results for removal efficiency for COD, TSS, NH_3_–N, and color were 53.50%, 60.26%, and 91.39%, respectively. The removal of selected heavy metals from leachate for Fe^2+^, Zn^2+^, Cu^2+^, Cr^2+^, Cd^2+^, Pb^2+^, As^3+^ and cobalt Co^2+^ were 89.76%, 94.61%, 94.15%, 89.94%, 17.26%, 93.78%, 86.43% and 84.19%, respectively. The results demonstrate that tannin-based natural coagulant could effectively remove organic compounds and heavy metals from stabilized landfill leachate.

## 1. Introduction

Landfilling is still considered a common and preferable method of disposal for solid municipal waste [1]. However, large quantities of leachate generated from landfills contain high levels of organic and non-organic pollutants, such as heavy metals, dissolved and colloidal solids and various pathogens that can potentially contaminate groundwater and surface water [2,3,4,5,6]. About 133 different toxic chemicals were discovered in 56 conventional municipal waste landfills compared to 72 toxic chemicals in industrial waste landfills [7]. In Malaysia, solid waste generation is expected to reach 30,000 tons per day in 2020, with a daily per capita average of 0.8 kg [8]. The production of solid waste in Malaysia is annually increased between 3% and 4% [9]. This increasing trend is due to rapid economic growth, rapidly changing life styles and rural-urban migration. Approximately 70% of this waste is collected and roughly 95% of collected waste is disposed in landfills [10]. As most of the dumpsites are old, the leachate produced has been stabilized and has low biodegradability [11,12,13]. Therefore, advanced treatment methods are necessary prior to discharge. Several treatment applications have been applied to leachate, such as coagulation and electrocoagulation [14,15], Fenton, photo-Fenton and Electro-Fenton reaction [16,17,18,19], ozonation-based advanced oxidation processes [20,21,22], adsorption and ion exchange [23,24]. In spite of the potential or promise demonstrated by certain treatment processes; questions about treatment cost, sludge production and chemical residues in treated effluent remain unanswered. The use of low cost and natural materials for wastewater treatment has recently gained increasing attention from a number of researchers. In the past two decades, many studies have been conducted to evaluate the effectiveness in substituting chemical coagulants with natural polymers in coagulation processes; polymers have been selected due to a number of advantages: the lower volume of biodegradable sludge generated from their use, their lower toxicity to the environment [25], and their ability to work without requiring pH adjustment for wastewater treatment [26]. Tannins contain positively charged organic compounds with a long polymer chain and can be used as a natural coagulant for industrial wastewater treatment [27]. Tannin is a class of pollutant recognized by its ability to precipitate pollutants of proteins [28].

Application of tannin and its effectiveness for the treatment of low organic water and wastewater has already been demonstrated [29,30,31]. However, few studies have investigated the use of tannin for stabilized leachate treatment. In the present work, the efficacy of tannin-based natural coagulants used for leachate treatment was investigated. Experimental conditions for pH and tannin dosage were optimized, and the removal abilities of the various treatments were determined. Moreover, the efficacy of tannin in removing heavy metals from leachate was also examined.

## 2. Methodology

### 2.1. Sampling and Site Characteristics

Leachate samples were collected from Ampar Tenang Closed Landfill Site (ATCL). This landfill site is located in the Sepang district, approximately 4 km to the southeast of Dengkil town in Selangor, Malaysia at latitude 02°48.925′ N and longitude 101°4.933′ E, 40 km southeast of Kuala Lumpur [32]. The landfill site is bound mainly by oil palm plantations, and housing projects have been developed closed to the landfill. The southern area of the site is located approximately 300 m from Labu River. The average precipitation in Dengkil is approximately 2450 mm per year. Annual temperatures consistently range from 24 to 32 °C with a mean temperature of 27 °C [33]. The ATCL is located on the Langat basin alluvial aquifer. Layers from silt and sands represent the shallow confined aquifer; however, the ground surface is more clayey [34], with thickness ranging from 5 to 12 m [35]. There are other layers of clay under the aquifer with thickness ranging between 8 and 15 m that results in the aquifer consisting of lenses on its bottom [36].

The ATCL has a total area of 10 acres. It has been operating for 15 years since 1994. During operation, ATCL received approximately 100 tons of solid waste per day. A total of 500,000 tons of solid waste has been disposed of at the site. The site was fully closed in 2010. ATCL was upgraded from dumping site (Level 0) to sanitary classification (Level 1) before it closed. Leachate samples were collected three times during the August 2017 and January 2018 period. Leachate was manually collected and placed in 500 mL polyethylene containers. The samples were immediately transported to the laboratory and cooled at 4 °C to reduce the biological and chemical reaction. The general characterization for leachate is presented in Table 1.

### 2.2. Tannin Characterization

**Figure 1:**
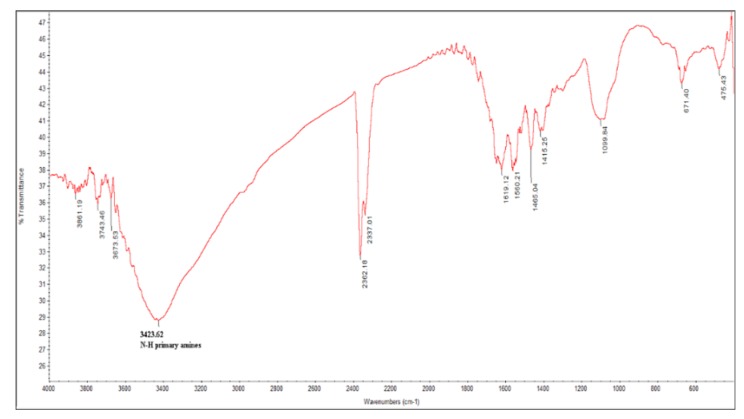
Fourier transformed infrared (FTIR) spectroscopy curve for modified tannin with amino group.

The tannin used in the present study is a commercial variety extracted from the bark of the Black Acacia (*Acacia mearnsii*) and modified by ammonium [27]. Tannins are hydrolyzable and/or condensed materials [39]. The tannin has a polymeric structure and contains amino groups [28], which are involved in the bridging mechanism used by particles during the coagulation process (Figure 1). The composition and physical properties of naturally extracted tannins are described by Roux et al. [40]. The phenolic building blocks substitution and the aliphatic hydroxyl groups enhanced the reactivity of the natural tannin [40,41,42]. The chemical modification of naturally extracted tannin was reviewed by Arbenz and Avérous [42]. Matamala et al. [43] improved the reactivity of natural tannins extracted from Chilean radiata pine species and from Brazilian black acacia using AISI 1010 (UNS G10100) steels.

### 2.3. Experimental Procedure

The experimental procedure was carried out in two stages; the first stage is a preliminary experiment, which was run using one factor a time to identify the region of interest for each influential variable (factor) for pH and tannin dosage and to select the appropriate levels. The selected levels for pH and tannin dosage were used to carry out the second stage using response surface methodology (RSM). A central composite design with two factors pH and tannin dosage and four responses were carried out.

#### 2.3.1. Effect of Tannin Dosage and pH

For the first stage; modified tannin was utilized for the coagulation of stabilized leachate. The different dosages of tannin ranging between 0.25 g and 1.25 g were added separately as a powder form to the 500 mL leachate samples. During this stage of experiment, the initial pH for the leachate sample (8.5) was kept without adjustment, and coagulation was evaluated based on chemical oxygen demand (COD), color, total suspended solids (TSS), and NH_3_–N removal efficiency. Maintaining the optimum dosage obtained from the previous stage, the influence of pH variation (ranging between three and 12) on the removal of targeted parameters was analyzed. For pH adjustment, 3M of hydrochloric acid solution and 3M of sodium hydroxide solution were used. The pH of all samples was adjusted to the desired value before coagulant addition. Prior to the coagulation process, leachate samples were thoroughly shaken to avoid the possibility of settling solids. The jar test is a method that uses different coagulant dosages to simulate coagulation to determine the minimal dosage required to obtain the highest removal efficiency for targeted parameters. The jar test was first conducted at 250 rpm for 15 min, which was then followed by 60 rpm for 30 min. Then, the liquor was left for 30 min to settle. After settling, the efficacy of various tannin dosages at removing targeted parameters was determined [44].

The removal efficiency for COD was calculated using the following equation, Equation (1):

Removal (%) = [(*C*_i_ − *C*_f_)/*C*_i_] × 100 (1)

where *C*_i_ and *C*_f_ are the initial and final COD concentrations.

#### 2.3.2. Optimization of Treatment Efficiency

A central composite design (CCD) for the tannin-based leachate treatment was created using design expert software (version 6.0.7) to investigate whether COD, color, NH_3_–N and TSS were influenced by pH values and the various dosages of tannin. The quantities and levels of each variable (factor) were selected based on the preliminary experiments explained in Section 2.3. Thirteen experiments were performed to cover all possible combinations between pH levels and tannin dosages. The levels of the selected factors (pH and tannin) in terms of actual and coded forms are provided in Table 2.

The data obtained from various experiments of CCD are usually used to fit a polynomial model and most probably a second-order model (see Equation (2)):

(2) $$Y=\beta_{0}+\sum_{j=1}^{k}\beta_{j}X_{j}+\sum_{j=1}^{k}\beta_{jj}X^{2}{}_{j}+\sum_{i}\sum_{<j=2}^{k}\beta_{ij}X_{i}X_{j}+e_{i}$$

where *Y* is the response, *X*_i_ and *X*_j_ are the variables, *β* is the regression coefficient, *k* is the number of factors studied and optimized in the experiment, and *e* is the random error. A *p*-value less than 0.05 was considered significant.

### 2.4. Analytical Study

The biochemical oxygen demand (BOD_5_), chemical oxygen demand (COD), ammoniacal nitrogen (NH_3_–N), total suspended solid (TSS), electrical conductivity (EC), pH and heavy metals—copper (Cu^2+^), iron (Fe^2+^), lead (Pb^2+^), and zinc (Zn^2+^)—were determined before and after each run of coagulation. The concentration of BOD_5_ was determined using Method 5210B. The dissolved oxygen (DO) was measured using a DO meter (model 1000, YSI Inc., Greene County, OH, USA). The COD concentration was determined using the closed reflux colorimetric method (5220B-DR2500 HACH, Loveland, CO, USA). Color concentration was measured using the DR 2800 HACH spectrophotometer at 455 nm wavelength. The pH and EC were measured using a portable digital pH/mV meter (model inoLab pH 720, WTW, Weilheim, Germany). Total suspended solids (TSS) was measured using method 2540C. NH_3_–N concentration was measured by the phenate method (4500-NH_3_ F) using a DR2500 spectrophotometer at 640 nm. Heavy metals were analyzed using Atomic Absorption Spectroscopy (Unicam 929 AA Spectrophotometer, UNICO, Franksville, WI, USA). All parameters were determined following standard methods for examination of water and wastewater [45]. Different tannin dosages (0.25, 0.50, 0.75, 1.00 and 1.25 g) were added to raw leachate in the beakers to evaluate its performance in removing targeted parameters.

## 3. Results and Discussion

### 3.1. Effect of Tannin

**Figure 2:**
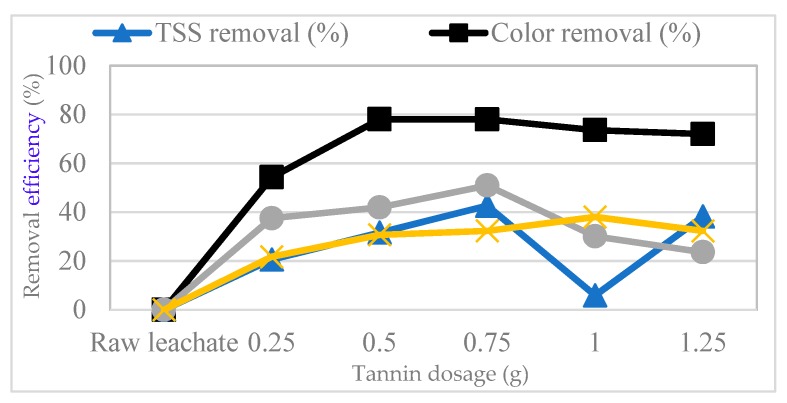
Effect of tannin dosage on chemical oxygen demand (COD), color, total suspended solids (TSS) and NH_3_–N removal from leachate at a pH of 8.18, 250 rpm for 15 min, 60 rpm for 30 min and settling for 30 min.

The effects of the various tannin dosages on the leachate samples were investigated. The various tannin dosages ranged between 0.25 g and 1.25 g. Its effects on COD, color, NH_3_–N and TSS removal were evaluated, as seen in Figure 2. The maximum removal percentage for COD, color and TSS were 54%, 78% and 43%, respectively at 0.75 g of tannin. For ammonia, higher removal was achieved using 1 g of tannin. The removal of targeted parameters was improved when tannin dosage increased between 0.25 g to 0.75 g. The removal of organic pollutants improved due to the ability of natural polyphenols in tannin to adsorb organics and metal ions [46]. The improvement in the removal of organic and ammonia may be due to the effect of electric double layers formed by carboxylic, phenolic and amino groups [47]. The removal efficiency of targeted parameters reduced when higher dosages of tannin (>0.75 g) were applied. The positively charged primary amino groups contained in tannin led to an improved bridging mechanism of the particles and colloids in the leachate and enhanced the flocculation process [27]. Tannin is unhydrolyzed in leachate and has a high molecular weight. The use of higher dosage of tannin leads to fast precipitation of large amount of tannin which may inhibit the flocculation efficiency [48].

### 3.2. Effect of pH Variation

**Figure 3:**
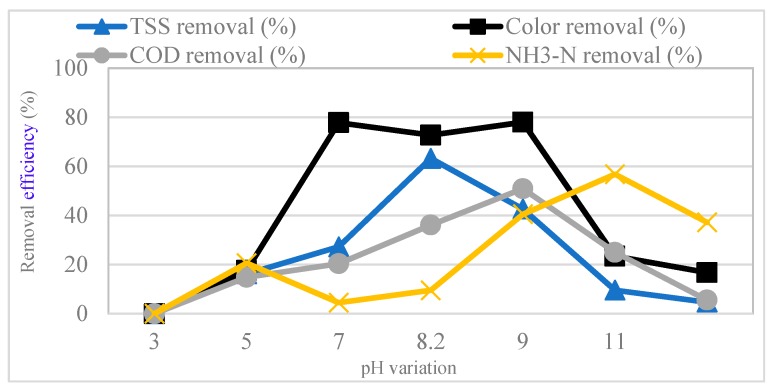
Effect of pH variation on COD, Color, TSS and NH_3_–N removal using a tannin dosage of 0.75 g at 250 rpm for15 min, 60 rpm for 30 min and after 30 min settling.

The effect of pH variation in the experiments on leachate coagulation by tannin was investigated by maintaining tannin dosage at 0.5 g. The pH levels used ranged between 3 and 11. Figure 3 shows that the maximum removal for COD (51%) and color (78%) was achieved at pH 9, while the highest removal from TSS (57%) and NH_3_–N (57%) was achieved at pH 8.18 and 9, respectively. At pH ranging between 7 and 9, the adsorption capability of the particles will be high due to the neutral electric charge [49]. Cations in leachate can improve the coagulation process by neutralizing and destabilizing the negative charges of the residue of the coagulant functional groups by linking with tannin particles [50]. The concentration of monovalent and multivalent cations in leachate, including Mg^2+^, Ca^2+^, Na^+^ and Fe^2+^, stimulated flocculating activity. These results are analogous to those uncovered by Okaiyeto et al. [51], Wang et al. [52] and Zhang et al. [53], who reported that the multivalent of cations such as Ca^2+^, Mn^2+^ and Al^3+^ increased flocculation activities. Cosa et al. [54] and Nwodo et al. [55] also reported increasing flocculating activity as a result of the influence of Ca^2+^, Mg^2+^ and Mn^2+^.

### 3.3. Scanning Electron Microscopy (SEM) Imaging

**Figure 4:**
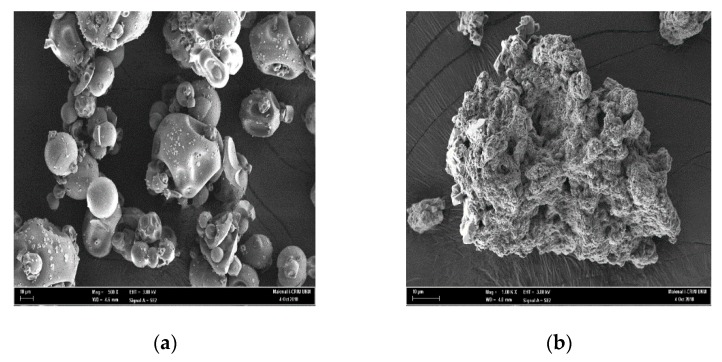
Microscopic image (10 µm) for tannin (**a**) before (**b**) after coagulation process observed by scanning electron microscopy.

The morphological surface structure of tannin was observed prior to and after the coagulation process. As shown in Figure 4a, tannin has a condensed crystalline brick-shaped structure. The structure served as an attachment site to which suspended particles and cations could bind [56]. Figure 4b illustrates how the coagulant aggregated the particles, which resulted in the formation of larger flocs that were easily settled. Therefore, SEM images of tannin indicated that bridging could be responsible for tannin’s impressive coagulation capabilities [56,57].

### 3.4. Analysis of Variance

**Figure 5:**
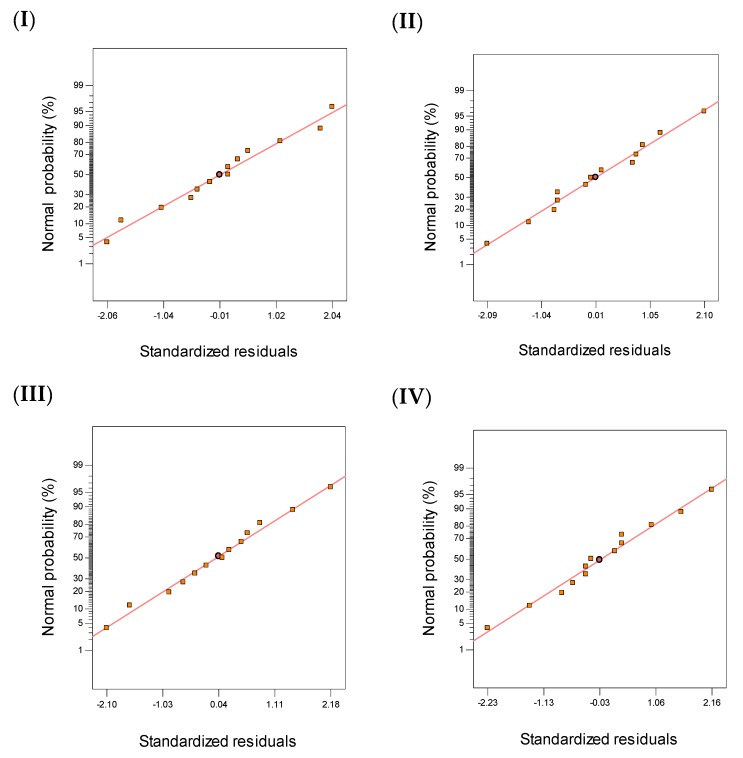
Normal probability plots for (**I**) COD, (**II**) color, (**III**) NH_3_–N and (**IV**) TSS removals from leachate.

The effect of pH and tannin dosages on four responses COD, color, NH_3_–N, and TSS was investigated using central composite design (CCD). The results of the 13-run (Table 3) obtained from CCD were analyzed using analysis of variance (ANOVA). The normality assumption for the data, which should be checked before starting the analysis, demonstrated that the data for all responses followed normal distribution as presented in Figure 5. The two variables, pH and tannin dosage, showed a significant effect (*p*-value < 0.05) for the linear or quadratic or both on the selected responses as presented in Table 4 for ANOVA results. Furthermore, the interaction effect between pH and tannin displayed a significant effect on color and TSS removal, which indicates that the two variables pH and tannin dosages do not work independently, while failing to show any significant effect on other removals. This means that the two variables work independently.

The effect of pH and tannin was modelled using a second-order model as given in Equations (3)–(6).

COD removal (%) = +35.88 − 13.30 × A − 9.79 × B + 3.34 × A^2^ − 6.93 × B^2^ + 1.30 × A × B (3)

Color removal (%) = +81.09 − 16.46 × A + 2.22 × B − 5.47 × A^2^ − 9.34 × B^2^ − 3.93 × A × B (4)

NH_3_–N removal (%) = +69.83 + 3.78 × A + 1.90 × B + 3.36 × A^2^ − 6.12 × B^2^ + 0.96 × A × B (5)

TSS removal (%) = +56.55 3.80 × A − 2.30 × B 0.64 × A^2^ − 7.64 × B^2^ + 4.08 × A × B (6)

**Figure 6:**
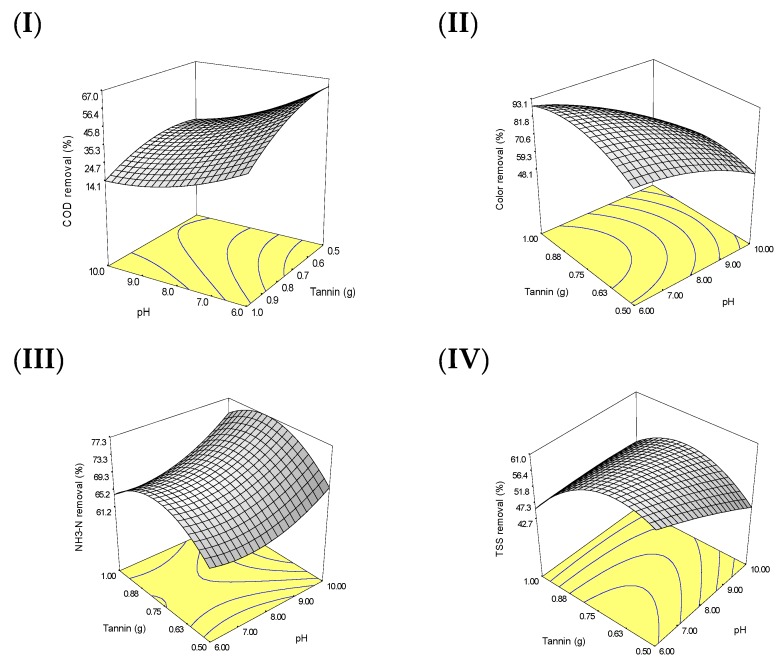
Response surface plots for (**I**) COD, (**II**) color, (**III**) NH_3_–N and (**IV**) TSS removals from leachate.

**Figure 7:**
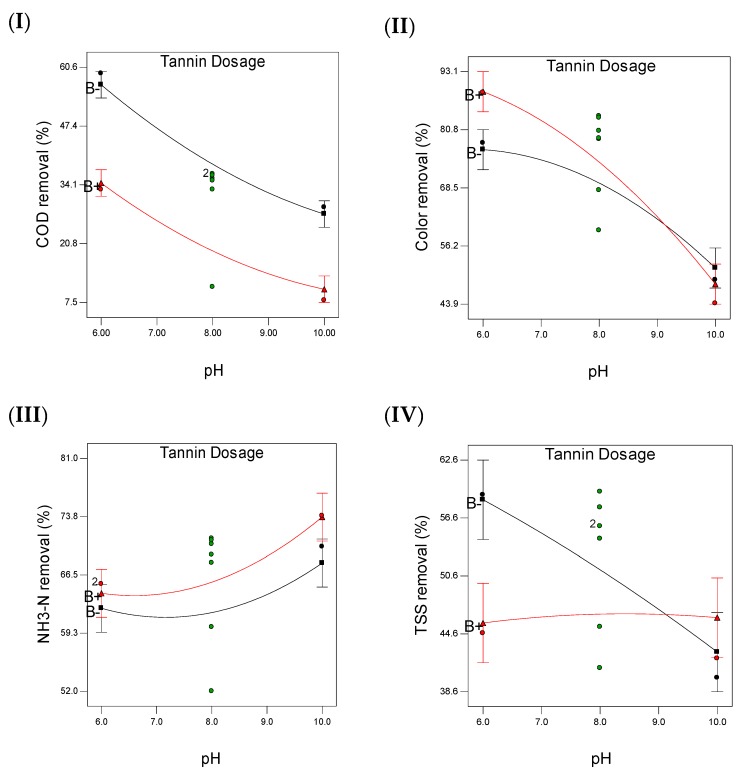
The effect of Interaction between tannin dosage and pH for: (**I**) COD, (**II**) color, (**III**) NH_3_-N and (**IV)** TSS removal during coagulation of landfill leachate (B−: represents the low level of tannin dosage, B+: represents the high level of tannin dosage and: represents the design point).

The models in Equations (3)–(6) are good because the coefficient of determination is high and close to 1 (Table 4), which indicates that most of the variance in the data is captured by the model. However, Equation (3) is not as good as other models in explaining the variance in the model since the lack of fit is significant (*p* < 0.003) which indicates that the model of COD is not as good as other models for color, NH_3_–N and TSS removals. This means that a higher order model such as third-order polynomial model or more complicated models can be used to enhance the model of COD removal. The effect of pH and tannin on the selected responses is pictorially presented in Figure 6. Figure 6 displays a three-dimensional response surface plot to describe the behavior of each response regarding pH and tannin. Figure 6 demonstrates the maximization of all four responses (COD, color, NH_3_–N and TSS); the region of maximum effect is well defined with the selected boundaries of pH and tannin. Figure 7 represents the interaction between the two main factors (pH and tannin) and their removal behaviors on the selected parameters.

### 3.5. Optimization of Leachate Treatment Using Tannin

The optimization process was carried out to determine the optimum value of COD, color, NH_3_–N, and TSS removal efficiency, using the Design Expert 6.0.7 software. According to the software optimization step, the desired goal for each operational condition (tannin dosage and pH) was chosen “within” the range. The responses (COD, color, NH_3_–N, and TSS) were defined as maximum to achieve the highest performance. The program combines the individual desirability into a single number, and then searches to optimize this function based on the response goal. Accordingly, the optimum working conditions and respective percentage removal efficiencies were established, and the results are presented in Table 5. As shown in Table 5, the removal for COD (53.50%), color (91.4%), NH_3_–N (69.7%) and TSS (60.7%) are predicted, respectively. The desirability function value was found to be 0.844 for these optimum conditions.

A confirmatory laboratory experiment was run using these optimum conditions, and the results obtained were the following: 60.30% TSS, 90.70% color, 52.80% COD and 66.70% NH_3_–N removal rates. The residual for COD, color, NH_3_–N and TSS was reported as 418 mg/L, 279 Pt-Co, 180 mg/L and 17 mg/L, respectively, which are still higher than discharge limits, except for TSS.

### 3.6. Heavy Metal Removal

The metal complexation of tannin is played an important role for heavy metal deposition from wastewater treatment using natural modified tannin [58]. Slabbert [59] characterized and identified five metal complexes formed by iron (Fe^+2^), aluminum (Al^+3^), titanium (Ti), and molybdenum (Mo) ions. The metal complexes increased the charged transfer and enhanced the adsorption and deposition of dissolved heavy metals in water [59,60,61,62,63]. The removal of selected heavy metals from leachate under the obtained optimum experimental conditions (tannin dosage 0.75 g, pH 6, 250 rpm for 15 min followed by 60 rpm for 30 min and settling for 30 min) was investigated, and the results are presented in Table 6. The removal efficiency for the majority of heavy metals ranged between 84% and 94%, while the lowest removal efficiency was reported for Cd^2+^ at 17.26%, which may be due to the optimal pH value for Cd removal being 5 [64], while the experiment for heavy metals removal was performed at pH 6. Tondi et al. [65] used tannin based rigid foam to enhance the removal of dissolved heavy metals in water. Oo et al. [66] employed mangrove tannins for removing lead (Pb^2+^) and copper (Cu^2+^) involving the ion exchange and complexation process by interaction of metals with hydroxyl groups.

## 4. Conclusions

In this study, the optimization of COD, TSS, color, and NH_3_–N removal for the coagulation treatment process of stabilized leachate was investigated. In the runs, the highest COD, TSS, color and NH_3_–N removal rates were achieved at 52.5%, 53.5%, 91.39% and 64%, respectively. Variables such as tannin and pH were modeled with satisfactory degrees of fit. The results suggest that a tannin dosage 0.73 g at a pH of 6 and duration of 45 min can be considered as an efficient pre-treatment process for leachate, and additional post treatment can be considered for further organic and ammonia removals.

## Figures and Tables

**Table 1:** Descriptive statistics including the mean and standard deviation of leachate quality parameters and heavy metals.

Parameter	Mean and Standard Deviation	(USEPA [37]; DOE [38])
pH	7.9 ± 0.5	6–9 **
EC (µS/cm)	6565 ± 324	-
TDS (mg/L)	4671 ± 174	-
TSS (mg/L)	40 ± 8	88 *
COD (mg/L)	893 ± 202	400 **
BOD_5_ (mg/L)	59 ± 10	20 **
NH_3_–H (mg/L)	531 ± 22	5 **
DO (mg/L)	5 ± 2	10 *
Mg (mg/L)	20 ± 4	-
Ca (mg/L)	40 ± 3	-
Na (mg/L)	639 ± 303	-
Fe (mg/L)	0.8 ± 0.2	5 **
Zn (µg/L)	280 ± 2	2000 **
Cu (µg/L)	42 ± 4	20 **
Cr (µg/L)	45 ± 2	10 **
Cd (µg/L)	0.6 ± 0.1	10 **
Pb (µg/L)	4 ± 1	10 **
As (µg/L)	17 ± 7	50 **
Co (µg/L)	11 ± 8	50 **
Mn (µg/L)	61 ± 49	20 **

* [37], ** [38].

**Table 2:** The pH levels and tannin dosages for central composite design (CCD) with axial points.

Level of Value			Tannin Dosage (g)		pH	
Tannin	pH	Coded	Actual	Coded	Actual	Coded
0.4	5.17	−1.414	0.5	−1	6	−1
		0	0.75	0	8	0
1.1	10.83	+1.414	1	1	10	1

**Table 3:** Experimental design conditions and actual and predicted removals for the parameters for leachate coagulation with tannin.

Run No.	Point Type	Factor 1: pH	Factor 2: Tannin (g)	COD Removal (%)			Color Removal (%)			NH_3_–N Removal (%)				Total Suspended Solids (TSS) Removal (%)	
				Actual	Predicted	Residual	Actual	Predicted	Residual	Actual	Predicted	Residual	Actual	Predicted	Residual
1	Axial	5.17	0.75	67.00	66.90	0.10	92.00	93.43	−1.43	68.90	71.20	−2.29	60.26	60.64	−0.38
2	Axial	10.83	0.75	28.00	27.60	0.40	51.00	46.87	4.13	81.00	81.88	−0.88	54.00	49.88	4.12
3	Center	8.00	0.75	37.50	36.34	1.16	78.81	81.09	−2.27	70.32	69.83	0.49	55.70	56.55	−0.85
4	Axial	8.00	0.40	34.00	37.69	−3.69	59.49	59.26	0.23	52.00	54.91	−2.91	45.28	44.52	0.76
5	Center	8.00	0.75	36.80	36.34	0.46	83.70	81.09	2.62	71.02	69.83	1.20	55.70	56.55	−0.85
6	Fact	10.00	0.50	31.00	28.57	2.43	49.00	51.52	−2.52	70.00	67.98	2.02	40.00	42.67	−2.67
7	Center	8.00	0.75	36.17	36.34	−0.16	79.00	81.09	−2.09	70.79	69.83	0.96	54.40	56.55	−2.15
8	Fact	6.00	1.00	33.00	35.93	−2.93	88.71	88.88	−0.17	65.37	64.22	1.15	44.63	45.69	−1.06
9	Fact	10.00	1.00	11.00	14.14	−3.14	44.00	48.10	−4.10	73.85	73.70	0.15	42.00	46.25	−4.25
10	Axial	8.00	1.10	13.00	8.81	4.19	68.00	65.53	2.47	60.00	60.27	−0.27	41.00	38.02	2.98
11	Fact	6.00	0.50	65.00	62.36	2.64	78.00	76.59	1.41	65.37	62.35	3.02	58.96	58.45	0.51
12	Center	8.00	0.75	35.04	36.34	−1.30	83.35	81.09	2.27	69.00	69.83	−0.83	59.28	56.55	2.74
13	Center	8.00	0.75	36.17	36.34	−0.16	80.56	81.09	−0.53	68.00	69.83	−1.83	57.65	56.55	1.11

**Table 4:** Analysis of variance (ANOVA) for COD, total suspended solids (TSS), color and NH_3_–N removals.

	**Source**	**Sum of Squares**	**DF**	**Mean Square**	**F Value**	**Prob > F**
**COD Removal (%)**	Model	2650.63	5	530.13	103.36	<0.0001
	A	1414.38	1	1414.38	275.77	<0.0001
	B	767.20	1	767.20	149.59	<0.0001
	A^2^	77.79	1	77.79	15.17	0.0059
	B^2^	334.50	1	334.50	65.22	<0.0001
	AB	6.81	1	6.81	1.33	0.2870
	Residual	35.90	7	5.13		
	Lack of Fit	34.46	3	11.49	31.87	0.0030
	Pure Error	1.44	4	0.36		
	Cor Total	2686.53	12			
	Std. Dev: 2.26, R^2^: 0.9866, Mean: 33.67; Adj R^2^: 0.9771, C.V: 6.73; Pred R^2^: 0.9079; Adeq Precision: 34.585					
**Color Removal (%)**	**Source**	**Sum of Squares**	**DF**	**Mean Square**	**F Value**	**Prob > F**
	Model	3003.96	5	600.79	58.23	<0.0001
	A	2167.80	1	2167.80	210.10	<0.0001
	B	39.36	1	39.36	3.82	0.0917
	A^2^	207.87	1	207.87	20.15	0.0028
	B^2^	607.43	1	607.43	58.87	0.0001
	AB	61.68	1	61.68	5.98	0.0444
	Residual	72.22	7	10.32		
	Lack of Fit	50.44	3	16.81	3.09	0.1523
	Pure Error	21.78	4	5.44		
	Cor Total	3076.18	12			
	Std. Dev.: 3.21, R^2^: 0.9765, Mean: 71.97, Adj R^2^:0.9598, C.V.:4.46 Pred R^2^:0.8723, Adeq Precision: 21.336					
	**Source**	**Sum of Squares**	**DF**	**Mean Square**	**F Value**	**Prob > F**
**NH_3_–N Removal (%)**	Model	529.27	5	105.85	20.75	0.0005
	A	114.12	1	114.12	22.37	0.0021
	B	28.75	1	28.75	5.64	0.0493
	A^2^	78.35	1	78.35	15.36	0.0058
	B^2^	260.56	1	260.56	51.08	0.0002
	AB	3.71	1	3.71	0.73	0.4220
	Residual	35.71	7	5.10		
	Lack of Fit	29.08	3	9.69	5.85	0.0604
	Pure Error	6.62	4	1.66		
	Cor Total	564.98	12			
	Std. Dev.: 2.26, R^2^: 0.9368: Mean: 68.13, Adj R^2^: 0.8917, C.V.: 3.32: Pred R^2^: 0.6156, Adeq Precision: 17.580					
**TSS Removal (%)**	**Source**	**Sum of Squares**	**DF**	**Mean Square**	**F Value**	**Prob > F**
	Model	631.55	5	126.31	13.02	0.0020
	A	115.80	1	115.80	11.94	0.0106
	B	42.23	1	42.23	4.35	0.0754
	A^2^	2.88	1	2.88	0.30	0.6030
	B^2^	405.95	1	405.95	41.84	0.0003
	AB	66.69	1	66.69	6.87	0.0343
	Residual	67.91	7	9.70		
	Lack of Fit	53.14	3	17.71	4.80	0.0820
	Pure Error	14.77	4	3.69		
	Cor Total	699.46	12			
	Std. Dev.: 3.11, R^2^: 0.9029, Mean: 51.45, Adj R^2^: 0.8336, C.V.: 6.05, Pred R^2^: 0.4267, Adeq Precision:10.690					

A: pH B: Tannin dosages.

**Table 5:** Optimal Response results from model prediction and laboratory.

pH	Tannin Dosage (g)	TSS Removal (%)	Color Removal (%)	COD Removal (%)	NH_3_–N Removal (%)	Desirability
6.00	0.73	60.3	91.3882	53.5	69.7	0.844
Lab experiment		60.7	90.7	52.8	66.3	

**Table 6:** Effect of tannin on heavy metal removal (tannin dosage 0.73 g, pH 6, 250 rpm for15 min, 60 rpm for 30 min and 30 min of settling).

Heavy Metals	Initial Concentration in Leachate	Residual After Tannin Coagulation	Removal (%)
Fe (mg/L)	0.8 ± 0.2	0.1 ± 0.0	89 ± 2
Zn (µg/L)	280 ± 2	15 ± 1	94 ± 3
Cu (µg/L)	42 ± 4	2.5 ± 0.3	94 ± 2
Cr (µg/L)	45 ± 2	5 ± 1	90 ± 1
Cd (µg/L)	0.6 ± 0.1	0.5 ± 0.1	17 ± 1
Pb (µg/L)	4 ± 1	0.3 ± 0.0	94 ± 2
As (µg/L)	17 ± 7	2.4 ± 1	86 ± 1
Co (µg/L)	11 ± 8	1.7 ± 0.5	84 ± 2
